# Intravenous vs intraosseous adrenaline administration in cardiac arrest: A protocol for systematic review and meta-analysis

**DOI:** 10.1097/MD.0000000000023917

**Published:** 2020-12-24

**Authors:** Wei Zhang, Yi Liu, Jing Yu, Dongze Li, Yu Jia, Qin Zhang, Yongli Gao, Xiaoyang Liao

**Affiliations:** a Department of Emergency Medicine, Laboratory of Emergency Medicine, West China Hospital, and Disaster Medical Center; b School of Nursing; c Department of General Practice and National Clinical Research Center for Geriatrics, International Medical Center, West China Hospital, Sichuan University, Chengdu, China.

**Keywords:** adrenaline, cardiac arrest, intraosseous, intravenous

## Abstract

**Introduction:**

Cardiac arrest refers to the sudden termination of cardiac ejection function due to various causes. Adrenaline is an important component of resuscitation among individuals experiencing cardiac arrest. The adrenaline delivery method chiefly involved intraosseous infusion and intravenous access. However, the impact of different adrenaline delivery methods on cardiac arrest has been unclear in previous research. Thus, the present study aimed to synthesize the available evidence regarding intravenous vs intraosseous adrenaline administration in cardiac arrest.

**Methods and analysis:**

We will search PubMed, EMBASE, Cochrane Library, Wanfang, and China National Knowledge Infrastructure. As per the inclusion criteria, randomized controlled trials (RCTs) on adrenaline administration in cardiac arrest were selected. The primary outcome was prehospital restoration of spontaneous circulation (ROSC); the secondary endpoints were survival, favorable neurological outcome at discharge, and poor neurological outcome at ≥3 mon.

We plan to use the Cochrane Collaboration's tool for assessing the bias risk for RCTs. The Grading of Recommendations Assessment, Development and Evaluation approach will grade the certainty of the evidence for all the outcome measures across studies. RevMan 5.3.5 will be used for meta-analysis. If the heterogeneity tests show slight or no statistical heterogeneity, the fixed effects model will be used, in other cases, the random effect model will be used for data synthesis.

**Results and conclusion:**

This protocol will determine which epinephrine delivery method is the optimal in the management of cardiac arrest. Our findings will help clinicians and health professionals in making accurate clinical decisions about adrenaline administrations in cardiac arrest.

**Ethics and dissemination:**

Ethical approval was not required because this study was planned as a secondary analysis. The results will be disseminated in peer-reviewed publications, journals, and academic.

**INPLASY registration number:**

INPLASY202090100 (DOI:10.37766/inplasy2020.9.0100).

**Key points**

- The study aim is to comprehensively evaluate the evidence for the delivery mode of epinephrine in cardiac arrest and compare intraosseous with intravenous mode in terms of the survival rate and prognosis.
- This protocol will show which delivery mode of epinephrine is more effective for managing cardiac arrest.
- We planned to conduct a methodological and heterogeneity study of the subgroups and a sensitivity analysis to evaluate the stability of the results in the meta-analysis.
- The limitations of this protocol may be related to the fact that we only searched the literature for studies published in the Chinese and English languages.
- Furthermore, the sample size of the study may be insufficient, and the methodological quality of the eligible trials may be poor.

## 1 Introduction

Cardiac arrest refers to the sudden termination of cardiac ejection function due to various causes, such as the disappearance of arterial pulsation, severe hypoxia, ischemia, and metabolic disorder. If a rescue is not timely, patients can present with irreversible brain and other organ damage and can even die within 4 to 6 min. Hundreds of thousands of people experience cardiac arrest annually. The survival rate of in-hospital cardiac arrest is ∼20%,^[1–3]^ and that of out-of-hospital cardiac arrest is <10%.^[4,5]^High-quality cardiopulmonary resuscitation and early defibrillation are the keys in rescuing patients.^[6,7]^ However, even if the return of spontaneous circulation (ROSC) is achieved, <50% of patients can be discharged to their homes.^[8]^

Adrenaline can increase coronary artery and cerebral perfusion pressures during cardiopulmonary resuscitation because of its vasoconstriction effects,^[9]^ therefore, adrenaline is useful to improve the ROSC.^[10–12]^ In 2020, the guidelines of the American Heart Association's (AHA's) Cardiac Life Support (ACLS) recommended the early use of adrenaline in cardiac arrest patients.^[13]^

The adrenaline delivery method mainly included intraosseous infusion (IO) and intravenous (IV) access. IO is the process of injecting directly into the bone marrow to provide a non-collapsible entry point into the systemic venous system. The IV access may be difficult to implement owing to the reduced volume of the circulating blood, and the delay in adrenaline administration decreased the ROSC in cardiac arrest. Recent studies have shown that IV administration was associated with increased rates of ROSC, survival to hospital discharge, and superior neurological outcome, but compared to the IO administration of adrenaline, the presumed advantage was only slightly shorter with the IO route.^[14]^ However, some other studies have not supported this conclusion.^[15,16]^ Therefore, this conclusion on the optimal adrenaline delivery method is contradicts the current evidence. The AHA's ACLS 2020 guidelines recommended the establishment of IO access if obtaining IV access is not feasible or successful in patients with cardiac arrest.^[13]^ However, the clinical evidence level is weak (Class 2b, LOE B-NR), and few systemic reviews have assessed the optimal adrenaline delivery route for cardiac arrest patients. Hence, in this protocol, the aim is to systematically collect all available studies comparing IV and IO adrenaline administrations in patients with cardiac arrest. Moreover, to comprehensively compare the differences in survival rate and prognosis between these two administration methods in patients with cardiac arrest, a systematic review and meta-analysis will be conducted.

## 2 Methods and analysis

The protocol will be prepared according to the recommendations of the Preferred Reporting Items for Systematic Reviews and Meta-Analyses Protocols. It was registered on the International Platform of Registered Systematic Review and Meta-analysis Protocols (INPLASY202090100).

### 2.1 Eligibility criteria

#### 2.1.1 Types of studies

We only plan to include RCTs in this study about the different adrenaline delivery methods in cardiac arrest. Observational studies, case reports, and animal studies will be excluded.

#### 2.1.2 Types of participants

This review will consider all patients with cardiac arrest who received different adrenaline administrations. Regardless of country of origin, age, race, and gender, participants with cardiac arrest from any cause will be included. However, patients with pre-existing do-not-resuscitate orders and those with unclear data on outcome status and adrenaline administration route were excluded.

#### 2.1.3 Patient and public involvement

There is no patients nor public involved in this protocol for a systematic review and meta-analysis.

#### 2.1.4 Type of interventions

The intraosseous route of adrenaline administration will be used as the intervention. Those who received intravenous adrenaline administration can be used as controls. Moreover, patients who received adrenaline via the endotracheal route or experienced failed administration attempts via route or via more than one administration route will be excluded.

### 2.2 Types of outcome measures

**The primary outcomes:** ROSC.

**The secondary outcomes:** Short-term survival (survival to hospital admission) and midterm survival (survival to hospital discharge). Favorable neurological outcome at discharge (evaluated using either the Glasgow Outcome Scale,^[17]^ Cerebral Performance Category^[18]^ or Modified Rankin Scale^[19]^) and poor neurological outcome at ≥3 months.

### 2.3 Data sources and search strategy

We will search studies in PubMed, EMBASE, Cochrane Library, Wanfang, and China National Knowledge Infrastructure using titles and keywords. And the search has no language restrictions. The Boolean logic operator will be used to connect the retrieval words, mainly via computer retrieval, supplemented by manual retrieval. To prevent omission, the researchers will conduct a second expanded retrieval of the references of the retrieved studies. Table 1 shows the search strategy used for PubMed, and similar strategies will be applied for other electronic databases.

**Table 1:** Search strategy for PubMed.

Number	Search terms
1	Heart arrest
2	Cardiac arrest
3	Asystole
4	Cardiopulmonary arrest
5	Or 1–4
6	Intraosseous infusion
7	Intra-osseous infusion
8	Intra osseous
9	Intra-osseous
10	Intraosseous
11	Or 6–10
12	Intravenous
13	Intravenous infusion
14	Intravenous drip
15	Drip infusion
16	Or 12–16
17	Epinephrine
18	Adrenaline
19	4-(1-Hydroxy-2-(methylamino)ethyl)-1,2-benzenediol
20	Epinephrine acetate
21	Medihaler-Epi
22	Epinephrine hydrochloride
23	Adrenaline hydrochloride
24	Epitrate
25	Lyophrin
26	Epifrin
27	Epinephrine bitartrate
28	Adrenaline acid tartrate
29	Epinephrine hydrogen tartrate
30	Adrenaline bitartrate
31	Or 17–30
32	“5 and 11 and 16 and 31” or “2 and 11 and 31” or “5 and 16 and 31”

### 2.4 Data collection and analysis

#### 2.4.1 Selection of studies

**Figure 1:**
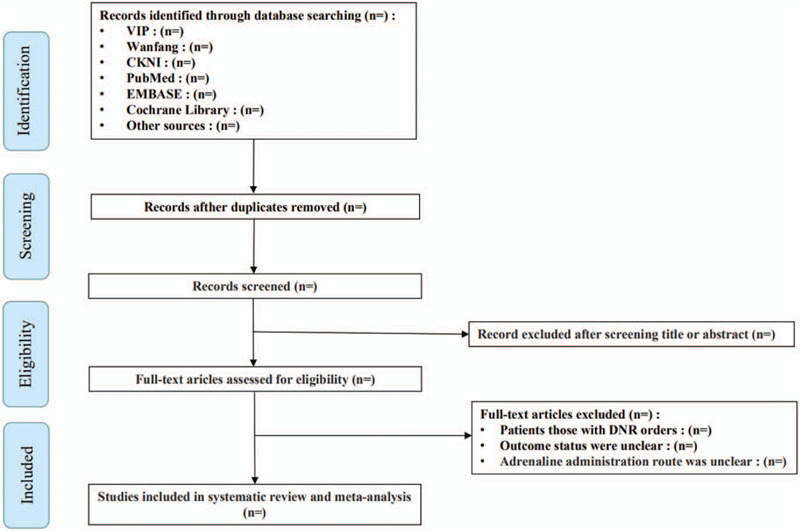
Flow diagram of this study selection. CNKI = China National Knowledge Infrastructure, DNR = do not resuscitate, VIP = Chinese Science and Technology Journal Database.

This study will use the Endnote X9.3.3 software to manage records. Two reviewers will independently evaluate all retrieved articles, conduct a preliminary evaluation of qualifications, eliminate duplicate articles and screen out ineligible studies such as incomplete articles, reviews and commentaries. Both parties, with the assistance of the third author, if necessary, will discuss and resolve inconsistencies via a re-evaluation of the original full text. The details of the study selection and identification process will be presented in a flow chart (Fig. 1).

#### 2.4.2 Data collection and management

After study selection, two independent reviewers will collect all essential data from each eligible study. The data are as follows: first author; year of publication, country where the study was conducted; the first author, year of publication, research background, age, race, gender, sample size, study methods, cause of cardiac arrest, adrenaline dosage, outcomes, and methodologic characteristics. The extracted findings from each paper will be checked consistency and consistency by the primary and secondary reviews. The reviewers will discuss and resolve any differences, and the third author will address the problem if necessary.

#### 2.4.3 Dealing with missing data

If there are unclear or missing data, we will contact the primary authors via E-mail. If there are no available data, we will impute the change scores with corresponding standard deviations, according to the Cochrane Handbook guidelines.

### 2.5 Study quality assessment

The Grades of Recommendation, Assessment, Development and Evaluation (GRADE)^[20]^ will be used by two independent reviewers to evaluate the quality of evidence. The GRADE includes the following domains: risk of bias, imprecision, inconsistency, publication bias and indirectness. GRADE evidence is categorised into four stages: high, moderate, low, and very low quality. The initial confidence level for each network estimate for randomised controlled trials is high. However, it will be degraded based on an evaluation using the five domains. The initial confidence level of each network estimate for observational studies is low. Nevertheless, it will be rated according to the three areas of assessment: large effects, specious mix and dose–response gradients.^[21]^ The GRADEprofiler software (GRADEpro V.3.6.1) will be used to complete the study quality assessment process.

### 2.6 Statistical analysis

#### 2.6.1 Outcome measures

The RevMan V.5.3.5 software will be used to conduct descriptive analyses. Descriptive statistics will be conducted to summarise the demographic and other relevant characteristics of the study population including each study cohort. For continuous variables, mean difference (MD) with 95% confidence intervals (CIs) will be used. For different measurement scales, the standardized MD (SMD) analysis with 95% CIs will be used. The dichotomous outcomes will be summarized as risk ratio (RR) or odds ratio (OR) with 95% CIs. All the analyses will be conducted based on the Cochrane Handbook for Systematic Reviews of Interventions.

#### 2.6.2 Assessment of heterogeneity

The presence of heterogeneity among the included studies is analysed using the chi-square test (test level: α = 0.1). The inconsistency index (*I*^2^) is used to evaluate heterogeneity, which represents the percentage of diversity, between studies. If *I*^2^ = 75% to 100%, will be considered as considerable heterogeneity; *I*^2^ = 50% to 90%, will be considered as substantial heterogeneity; and *I*^2^ = 30% to 60%, will be considered as moderate heterogeneity. If *I*^2^ = 0% to 40%, will be considered as no heterogeneity.

#### 2.6.3 Assessment of reporting bias

If sufficient RCTs (>10 RCTs) can be included, Begg's test and Egger's regression asymmetry test funnel chart will be used to evaluate the publication offset probability. If the probability of the Egger test is <10%, this indicates a publication bias among the studies.

#### 2.6.4 Data synthesis

The RevMan V.5.3.5 software will be used for data synthesis. If heterogeneity tests show slight or no statistical heterogeneity (*I*^2^ = 0% to 40%), the fixed effects model shall be used. If heterogeneity tests show significant heterogeneity (40% ≤ *I*^2^ < 75%), the random effect model will be used for data synthesis. If there is considerable heterogeneity in the study, the meta-analysis will not be performed. If there is clinical heterogeneity, we will conduct subgroup and meta-regression analyses. If the source of heterogeneity is not clear, a descriptive analysis will be conducted.

#### 2.6.5 Subgroup analysis

We will also conduct a subgroup analysis of rescue sites (in-hospital vs out-of-hospital rescue), adrenaline dosages (standard- vs high-dose adrenaline), causes of cardiac arrest, types of cardiac arrest (with a shockable rhythm vs nonshockable rhythm), gender, age (young age group, middle age group, older age group), country and outcome measures. In-hospital rescue is defined as the occurrence of cardiac arrest and rescue and the establishment of infusion channels in the hospital (ward or emergency). Out-of-hospital rescue is defined as the occurrence of cardiac arrest outside of the hospital, rescue via ambulance to the scene and establishment of the venous route at the scene or during transit. Moreover, an epinephrine dose of 0.1–0.2 mg/kg is considered high. Young age group is defined age < 18. Middle age group is defined 18 ≤ age < 60. Older age group is defined age ≥ 60.

#### 2.6.6 Sensitivity analysis

Sensitivity analysis will be conducted in order to evaluate the stability of the analysis results. The method is to delete the low-level quality research and then merge the data for evaluating the impact of the sample size, research quality, missing data, and statistical methods on the results of meta-analysis. However, if all the included studies include a high risk of bias, the sensitivity analysis will not be used.

### 2.7 Ethics and dissemination

A systematic review involves the secondary analysis of the published articles and does not require ethical approval. We will not endanger individual's privacy or impair their rights. The results will be disseminated in peer-reviewed publications, journals, and academic gatherings.

#### 2.7.1 Time line

This systematic review and meta-analysis will be finished by 2021 (Table 2 shows the detailed timeline of the project).

**Table 2:** Time line.

Task	Deadline
Protocol development	November 2020
Searches	January 2021
Abstract and full-text screening	March 2021
Data extraction	June 2021
Analysis	August 2021
Manuscript submission	November 2021

## 3 Discussion

The rapid establishment of the vascular route is important in the resuscitation process among critically ill patients.^[22–24]^ The peripheral IV route has been the traditional route for providing emergency pharmacotherapy. Previous studies have shown that the IO route is an excellent alternative to vascular delivery of liquids, blood products and drugs. Moreover, it can reduce the time in obtaining vascular access.^[25–27]^ The use of emergency pharmacotherapy administration via the IO route as a first-line approach for vascular access is becoming well-known in clinical settings. However, previous studies did not validate the importance of the adrenaline delivery route. Thus, this study will evaluate the advantages, disadvantages and efficacy of IO and IV administrations to help clinicians select the best adrenaline administration method and improve survival rate and prognosis in patients with cardiac arrest.

The strengths of this protocol are as follows. We will search comprehensive databases and perform more stringent and detailed quality assessment and data extraction. This systematic review and meta-analysis will use indirect and direct evidence to summarise and compare the effects of IV vs IO adrenaline administration in patients with cardiac arrest. However, the protocol still has several limitations. That is, the sample size may be small. The methodological quality of the eligible trials may be poor, and we only search for studies written in Chinese and English. However, this protocol can present the most effective epinephrine delivery method in the management of cardiac arrest and provide information that will be essential for the AHA guidelines.

## Acknowledgments

We would like to thank our subject librarians for their help with finalising the search strategy.

## Author contributions

WZ, DL, and XL conceived and designed the research; YL, YJ, and DL wrote the first draft; JY, QZ YG, and XL reviewed and contributed to drafting, revising, and finalizing the manuscript. All authors have reviewed and approved the final version of the manuscript and have given their permission for publication.

**Conceptualization:** Jing Yu, Dongze Li, Yu Jia.

**Investigation:** Qin Zhang, Yongli Gao.

**Methodology:** Yi Liu.

**Writing – original draft:** Wei Zhang, Xiaoyang Liao.

**Writing – review & editing:** Xiaoyang Liao.
